# Application of Commercial Biopreservation Starter in Combination with MAP for Shelf-Life Extension of Burrata Cheese

**DOI:** 10.3390/foods12091867

**Published:** 2023-04-30

**Authors:** Giuseppe Natrella, Giuseppe Gambacorta, Michele Faccia

**Affiliations:** Department of Soil, Plant and Food Sciences, University of Bari, Via Amendola 165/A, 70126 Bari, Italy

**Keywords:** burrata cheese, shelf life, protective culture starter, MAP, sensory analysis, chemical analysis

## Abstract

Burrata is a fresh pasta filata cheese manufactured in Italy. Its demand on the worldwide market is constantly growing, and prolonging its shelf-life is an important challenge for the Italian dairy industry. In the present study, combining a commercial bio-protective starter and modified atmosphere packaging (MAP) was evaluated as a strategy to delay the spoilage of product quality. Three experimental samples of burrata were produced by experimental trials at the industrial level and stored for 28 days under refrigerated conditions. Two samples contained the protective starter but were packaged differently (under MAP and immersed in water), and one did not contain the starter and was packaged under MAP. A sample of burrata without a starter and immersed in water was also prepared and used as a control. The combination of MAP and bio-protective starter delayed the degradation of lactose and citric acid, used as indices of microbial activity. In fact, lower counts of *Enterobacteriaceae* and *Pseudomonas* were observed in this sample. In contrast, control burrata had the highest level of total Volatile Organic Compounds (VOC) at the end of the storage period, because of higher microbial activity. Even though all samples were judged to be unacceptable after 28 days from the sensory point of view, the sample with bio-protective starter under MAP had the best score after 21 days, obtaining a shelf-life extension of about 7 days with respect to control. In conclusion, the combination of MAP and protective starter culture could be an easy way to extend the shelf-life of burrata stored under correct refrigerated conditions.

## 1. Introduction

Shelf-life extension of fresh food products is increasingly requested by manufacturers, retailers and consumers. For this challenging task, both conventional (i.e., thermal treatments, cooling, freezing, water activity reduction and use of antimicrobial molecules) and new technologies (i.e., high-pressure processing, pulsed electric fields, ultrasound, membrane filtration) and a combination of them, can be applied [1]. In general, products that suffer the most from short shelf life are those with high pH, water activity and fat content, as are fresh cheeses. For these dairy products, several innovative strategies have been proposed to improve preservability: use of natural bio-active preservatives such as endolysins, lysozyme, lactoferrin or essential oils [2,3,4,5,6,7]; strategies involving preserving brines [8,9,10,11]; non-thermal treatments such as high-pressure processing, pulsed light, ultrasonication and cold plasma [12,13,14,15,16]. Moreover, novel packaging systems (edible films and coatings), modified atmosphere packaging or protective cultures have been tested [17,18,19,20,21,22,23]. 

A fresh cheese that is becoming very popular worldwide is Burrata, an Italian pasta filata cheese with a “double-structured texture”. In fact, it is composed of an external pasta filata pouch filled with a cream called “stracciatella,” made of double cream mixed with thin strings of mozzarella. The chemical composition is characterized by high fat and moisture content and high pH; consequently, it is highly perishable due to the easy growth of microorganisms and oxidation [24]. For a long time, its consumption has been limited to the Apulia Region (Southern Italy), where it was developed at the beginning of the last century. The recent improvement in the hygiene conditions of the dairies and the microbiological quality of milk and cream led to better preservability. Still, efforts are continuously made to get further shelf life extension. To this aim, several technological solutions have been proposed, including the application of low temperatures during the whole processing phase [25], the use of protective cultures against spoilage bacteria [26], the use of modified atmosphere packaging (MAP) alone or in combination with lysozyme/EDTA disodium salt [27].

Moreover, a combination of antimicrobial molecules, active coating and MAP was tested by Costa et al. [28]. Other researchers aimed to reduce the fat content to lower the susceptibility to oxidation and, at the same time to improve the nutritional characteristics. Trani et al. [24] partially replaced fat with carob seeds flour and fortified the obtained cheese with polyunsaturated-fatty acids (PUFAs), Costantino et al. [29] used semi-skimmed milk and reduced fat-cream combined with carrageenan xanthan or exopolysaccharides produced by starters. In most of these studies, the results were not applicable in practice due to legal concerns, high costs or excessive variation of the organoleptic characteristics.

The present work aimed to evaluate an innovative and cheap solution applicable at an industrial scale to extend burrata shelf-life without using chemical preservatives. To this purpose, an approach not yet tested on this cheese, which combines a mixed protective starter culture with MAP, was tested. In particular, the investigation focused on the changes in the chemical and sensory characteristics of the cheese during refrigerated storage.

## 2. Materials and Methods

### 2.1. Sample Preparation

The cheese samples were prepared in a dairy in Andria (Apulia region, Southern Italy) by dedicated cheesemaking trials (three replicates). As burrata cheese can be sold while wrapped in flexible plastic bags (High-Density Polyethylene with 2 µm of thickness) or not wrapped and immersed in water packaged in trays sealed with plastic film, the experimentation considered both types. As MAP cannot be applied to the packages containing water, the experimental samples were prepared as follows:

- Wrapped burrata with protective starter packaged in MAP 70 N_2_% and 30% CO_2_ (coded as Ferm-MAP);
- Wrapped burrata packaged in MAP 70 N_2_% and 30% CO_2_ (coded as MAP);
- Water-immersed burrata with protective starter (coded as Ferm).

A burrata sample without the addition of a starter and immersed in water was used as a control (coded as Ctr). 

**Figure 1:**
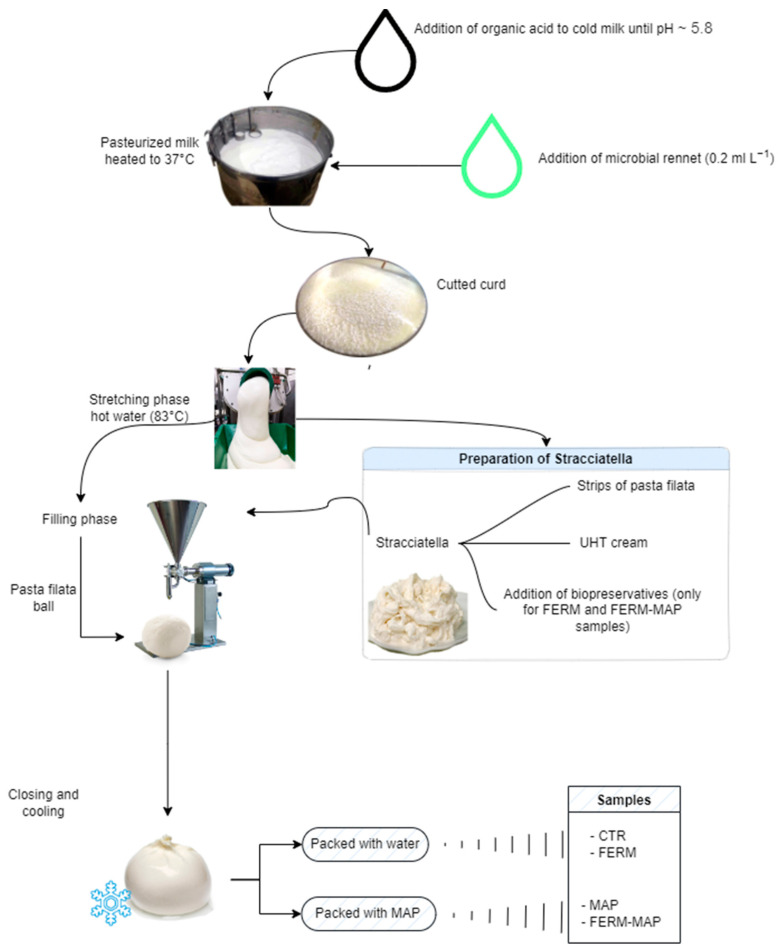
Experimental design of the burrata production.

Details about the experimental design are shown in Figure 1. Briefly, pasteurized milk was pre-acidified with lactic acid to pH 5.8 and then coagulated with 0.2 mL L^−1^ of microbial rennet (153 IMCU, pure chymosin, Sacco srl, Cadorago, Italy); an automatic cutter included in the industrial vat cut the curd (about 1–2 cm cubes) and whey drainage was subsequently done, the acidified curd was mechanically stretched and shaped in hot water (83 °C) to obtain a mozzarella ball. The warm ball was immediately transferred to a “blowing machine” that inflated it to form a pouch and then filled with stracciatella cream (previously prepared to mix UHT cream with freshly prepared mozzarella strands, 1:1 *w*/*w*). After the filling phase, the burrata was tied with a plastic strip and cooled down in chilled water. The protective starter culture was added to the experimental samples by inserting it into the UHT cream used to prepare stracciatella. It was a mixture of *Lactobacillus rhamnosus* (with probiotic function) and *Lactobacillus plantarum* (bacteriocin producer) supplied by Sacco srl (Cadorago, Italy). The samples (125 g weight) were stored in refrigerated conditions as indicated by the sector legislation for this type of dairy product (4 ± 2 °C) and were analyzed in duplicate at 0, 7, 14, 21 and 28 days after the manufacturing process. The sampling time went beyond the expiry date fixed by the manufacturer (14 days from production) to evaluate the effectiveness of the technological solution tested in extending shelf life.

### 2.2. Chemical Analyses

The lactose content was determined by following the method reported by Natrella et al. [25]. In brief, ten grams of blended samples were inserted in a 50 mL plastic falcon and added 20 mL of water, shacked for 1 h and then centrifuged at 4 °C × 6000 RCF × 10 min. The supernatant was filtered through a 0.2 µm syringe filter, and 10 µL aliquot was injected into the High-Performance Liquid Chromatography-Refractive Index Detectors system (HPLC-RID) (Agilent Technologies, Palo Alto, CA, USA) equipped with a Rezex RCM-monosaccharide Ca^2+^ column (300 × 7.8 mm, Phenomenex, Torrance, CA, USA) heated at 80 °C. The chromatographic runs were performed in isocratic conditions using deionized water as a mobile phase at 1 mL min^−1^ flow rate; the RID temperature was set at 40 °C. The identification and quantification of the lactose were made by using a reference standard of lactose (SIGMA-ALDRICH, Steinheim, Germany) and an external calibration curve.

Organic acids were separated, as reported by Natrella et al. [3]. The extraction was done as follows: 5 g of blended sample were added with 20 mL of 0.1% orthophosphoric acid in water, shacked for 30 min and then centrifuged at 4 °C × 5000 RCF × 15 min. The supernatant was filtered through a 0.2 µm syringe filter, and 20 µm aliquot was injected into the High-Performance Liquid Chromatography-Diode Array Detector system (HPLC-DAD) (Waters 996, Milford, MA, USA). A Synergi Hydro-RP80 (250 × 4.6 mm, Phenomenex Ltd., Aschaffenburg, Germany) column set to 30 °C was used for the separation. The mobile phase was composed of 0.1% orthophosphoric acid in water (A) and Acetonitrile (B); all solvents were HPLC-GRADE. The gradient was 0–18 min 100% A at 1 mL min^−1^ flow rate, then 18–18.3 min from 100% to 20% A; 18.3–19.5 min increasing flow rate to 1.4 mL min^−1^, then 19.5–22.5 isocratic and 22.5–23 min from 20% to 100% A held for 20 min in an isocratic condition. Detection was done at λ = 214 nm, and the results were expressed as mg g^−1^ of the sample by using external calibration curves of the analytes considered (SIGMA-ALDRICH, Steinheim, Germany).

Free fatty acids were extracted as reported by McCarthy et al. [30] and separated by a Gas Chromatography-Flame Ionization Detector (GC-FID) system (7890AGC-System, Agilent Technologies, Palo Alto, CA, USA). In brief, 2 µl of the extract was injected into the injector port, and the separation was performed using a HP5 column (30 m × 0.32 mm × 0.25 μm; Agilent Technologies). The oven parameters were: starting temperature 35 °C held for 1 min, then 15 °C min^−1^ until 75 °C; once reached, the temperature was held for 1 min, 3 °C min^−1^ until 90 °C, 20 °C min^−1^ until 180 °C; and finally, held at the isothermal temperature for 5 min. FID temperature was set at 220 °C. Free fatty acids were identified and quantified using standard calibration curves of the analytes considered (SIGMA-ALDRICH, Steinheim, Germany), and results were expressed as mg 100 g^−1^ of the sample.

VOC analysis was done, as reported by Natrella et al. [31]. The extraction technique used was Solid Phase Micro Extraction (SPME) with a divinylbenzene/carboxen/polydimethylsiloxane (DVB/CAR/PDMS) 50/30 mm SPME fiber assembly (Supelco, Bellefonte, PA, USA). The extraction was done by using a Triplus RSH autosampler installed on a GC-MS system (Thermo Fisher Scientific, Milan, Italy). One gram of the sample and 10 µL of the internal standard (3-pentanone, 81.2 ng) were inserted in a 20 mL glass vial and closed by a silicone/PTFE septum and aluminum cap, and then all vials were loaded into the autosampler. The equilibration time was 10 min at 37 °C, then the extraction was carried out at 37 °C for 15 min, during which the fiber was exposed to the vial headspace. The desorption phase was done into the injector port at 220 °C for 2 min by using the helium as carrier gas (99.9995% of purity, Nippon Gasses Industrial srl, Milan, Italy). The molecules were separated by using a VF-WAX MS thermo capillary column (60 m, 0.25 μm i.d., 0.25 mm, Agilent J&W) under the following conditions: oven temperatures 50 °C for 0.1 min, then 13 °C min^−1^ to 180 °C, 18 °C min^−1^ to 220 °C final isothermal for 1.5 min. The detector parameters were detector voltage 1700 V; source temperature 250 °C, ionization energy 70 eV and scan range 33–200 amu. Peak identification was made by means of Xcalibur V2.0 software, in particular Qual Browse (Thermo Scientific, Waltham, MA, USA), by matching with the reference mass spectra of the National Institute of Standards & Technology (NIST) library, while the semi-quantification was done by using the standard internal method.

### 2.3. Microbiological Analyses 

The counts of the most important spoilage and hygiene indicators bacteria were carried out. Ten grams of burrata and 90 mL of Butterfield’s phosphate-buffered water (Difco, Sparks, MD, USA) were inserted into a Stomacher bag and homogenized using a BagMixer stomacher (Interscience, St Nom, France). Then, serial dilutions were made of the homogenate and plated on the appropriate media in Petri dishes following the standard methods: *Enterobacteriaceae* [32]; *Pseudomonas* spp. [33]; *Escherichia coli* [34]; coagulase-positive *Staphylococci* [35] and Coliforms [36]. 

### 2.4. Sensory Analysis 

The analyses were performed by a trained panel composed of 5 experts belonging to the Italian Association of Cheese Tasters (ONAF) with at least five years of experience in the field and selected following ISO 8586:2012 [37]. The TCATA (Temporal Check All That Apply, a qualitative analysis) test is an expansion of the CATA (Check All That Apply) method; it was successfully used in a previous paper with a different approach compared to the typical use of this test, aiming to understand the differences among samples during their shelf-life [25]. The CATA approach (and its expansion) belongs to the flash profile methods used to quickly obtain a sensory profile and is commonly used in consumer science tests involving a high number of consumers. This method was originally used with trained panelists, then its popularity grew with consumers as judges for marketing purposes [38]. However, it has been demonstrated that results provided by the CATA test with a high number of consumers generate similar responses of a classical product characterization (i.e., Quantitative Descriptive Analysis) made by a trained panel [39,40]. For each analysis time (T0, T7, T14, T21 and T28), panelists were provided with a list of descriptors (previously selected in two preliminary sessions by expert panelists) from which they had to select all the words that apply and better describe the product. Sensory evaluations were conducted in a sensory laboratory equipped with individual cabins

### 2.5. Statistical Analysis 

The dataset was used to perform several analyses using XLSTAT (Addinsoft, Paris, France). Analysis of variance (ANOVA) was performed for chemical and microbiological data; Agglomerative Hierarchical Clustering (AHC) was used for VOC profile grouping, while Temporal Check All That Apply (TCATA) was performed for sensory analysis results.

## 3. Results and Discussion

**Figure 2:**
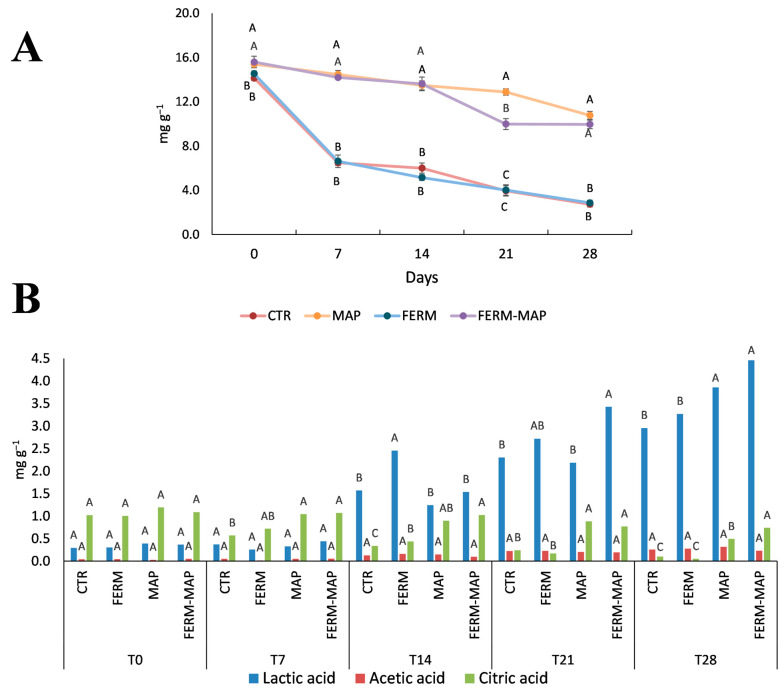
Lactose (**A**) and organic acid (**B**) content in burrata samples, each sampling time was considered separately. Ctr—control burrata; MAP—burrata packaged in the modified atmosphere; Ferm—burrata added microbial protection culture; Ferm-MAP—burrata added microbial protection culture and packaged in modified atmosphere. Results expressed as mg g^−1^ and statistically different at *p* < 0.05. Different letters indicate significant differences among samples considering each time separately (**A**). Different letters indicate significant differences among samples considering both time and acids separately (**B**).

The variations in the content of lactose, organic acids, FFA and VOC were used as chemical indices to monitor the decay process during cheese storage [3,41]. The lactose and organic acids contents are reported in Figure 2. As expected, lactose decreased during storage, but at different rates: the water-immersed samples underwent a dramatic decrease reaching the final concentrations of 2.59 (Ctr) and 2.72 mg g^−1^ (Ferm), whereas the MAP-packaged samples evidenced a slower drop with the final concentration of 10.63 mg g^−1^ (MAP) and 9.82 mg g^−1^ (Ferm-MAP). The better preservation of lactose in the MAP samples cannot be simply ascribed to an antimicrobial effect of the modified atmosphere since lactose tends to dissolve in the packaging water [41]. The samples packaged in water evidenced already at day 0 lower concentration with respect to the MAP samples (14.0 and 14.4 mg g^−1^ for Ctr and Ferm vs. 15.26 and 15.45 mg g^−1^ for MAP and Ferm-MAP). 

Further information is derived from the analysis of the organic acids (Figure 2B), whose formation is strictly related to the metabolism of microorganisms and consequently to the storage time; several authors have tried to classify cheese age by determining the organic acid profile [42,43,44]. Lactic acid is the main end-product of bacterial activity in all cheeses. It tends to decrease in long-stored/ripened types; other acids (acetic, formic, propionic and others) are secondary fermentation products. Instead, citric acid derives from milk and tends to disappear over time, being used as a fermentation substrate [45,46,47]. 

In detail, no differences among samples were observed in the lactic acid content until 2 weeks of storage, as refrigerated storage limited formation. Then, the concentration increased, and differences among samples became significant, with the samples packaged in MAP showing the highest level after 28 days. Concerning acetic acid, the results were similar to that reported by Faccia et al. [48] and Tirloni et al. [49]. Commonly, fresh products like burrata contain a small amount of this acid, and at low concentrations, its role in the sensory characteristics is negligible. The increase of this compound during storage was very slow, reaching the highest amount of 0.32 mg g^−1^ after 28 days in the MAP sample. Finally, citric acid decreased over time; the initial concentration ranged from 1.01 to 1.2 mg g^−1^, while at the end of the storage, it decreased to a different extent, resembling the behavior of lactose. In fact, the decrease was much more evident in water-immersed than in the MAP samples (0.06 and 0.1 mg g^−1^ in Ctr and Ferm vs. 0.50 and 0.74 mg g^−1^ in MAP and Ferm-MAP, respectively). Overall, organic acid solubilization in water played a role as they are also water-soluble.

**Figure 3:**
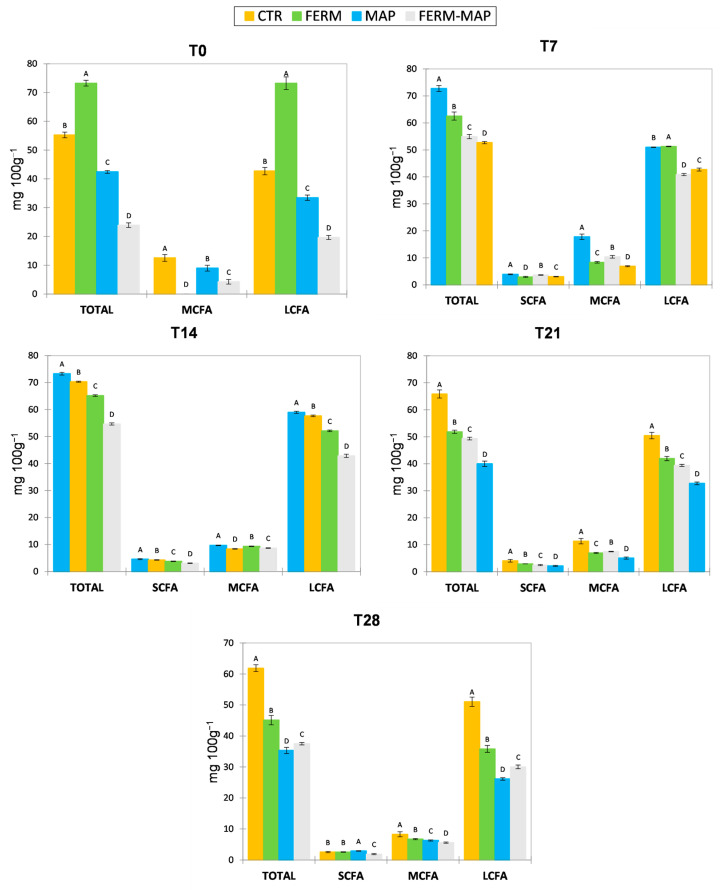
Free fatty acids content grouped by short, medium and long chain fatty acid (SCFA, MCFA and LCFA, respectively) and total amount found in burrata samples from T0 to T28. Ctr—control burrata; MAP—burrata packaged in the modified atmosphere; Ferm—burrata added of microbial protection culture; Ferm-MAP—burrata added of microbial protection culture and packaged in modified atmosphere. Results are expressed as mg 100 g^−1^ (*p* < 0.05). Different letters indicate significant differences among samples considering the total, SCFA, MCFA and LCFA separately at each time.

Figure 3 shows the content of free fatty acids (FFA), which is an index of lipolysis that, in cheese, is performed by lipases from milk, rennet paste (when used), and starter and non-starter microorganisms [50,51]. Differently from many other cheeses, lipolysis in Burrata is undesirable since FFA negatively affects the desired mild aroma. For this reason, rennet paste is not used in the manufacturing process, and curd acidification for stretching is not obtained by lactic starter fermentation but by direct milk acidification. Normally, these technological conditions, together with refrigerated storage and the short time from production to consumption, do not allow the significant breakdown of triglycerides into free fatty acids [25]. In the present experimentation, the addition of a protective starter represented a new variable that required monitoring of lipolysis. The obtained results indicated a delay of lipolysis in the three experimental samples compared to the control, with the combination MAP + protective culture showing the best result, probably as a consequence of a synergic effect. The effect was much more evident after 14 days, whereas a fluctuating trend of FFA concentration was observed in the first stages of storage. It was probably due to the concomitant formation and conversion of FFA to other compounds, as reported by several authors [52,53]. The Ferm sample had the highest amount of FFA at day 0, mostly ascribable to the increased presence of free palmitic acid. Then, the MAP sample showed the highest total FFA concentration between 7 and 14 days of storage, also in this case, due to the palmitic acid level. From 21 to 28 days of storage, Ctr evidenced the highest formation of volatile and semivolatile FFA (butyric, caproic, caprylic, capric and myristic) that are known to negatively affect the sensory characteristics of burrata, and almost twice the higher content of total FFA compared to the three experimental samples. Considering the sum of short, medium and long-chain fatty acids (SCFA, MCFA and LCFA), all samples shared the absence of SCFA and the presence of LCFA at day 0; it is worth highlighting that LCFA does not impact any flavor, since they have high odor perception threshold [54]. After 7 days, SCFA became detectable in all samples, even though the concentrations of butyric, caproic and caprylic acids were very low and far from levels able to alter the sensory characteristics. After 14 days of storage, the MAP and Ctr samples had higher amounts of SCFA and LCFA than the samples added with protective cultures, but successively, the situation was reversed, and a significant effect of MAP in delaying lipolysis was observed until the end of the storage.

The observed chemical changes must be interpreted in the light of the storage conditions applied, since refrigeration is known to favor psychrotrophic bacteria that are highly lipolytic [55,56]. Table 1 shows the evolution of the counts of the five bacteria groups considered over time.

In general, the microbial quality of the samples was very good in comparison to the count values reported in the literature [27,28,57]. On fresh burrata samples, the highest counts were found in Ctr and MAP for *Enterobacteriaceae*, Coliforms and *Pseudomonas*, with values ranging from 2.0 to 2.3 log CFU g^−1^. Probably an immediate control of the starter on these bacteria was observed, limiting their growth in the early storage phase. In addition, the combination of starter and MAP showed the lowest counts (1.5 to 1.9 log CFU g^−1^ for these three microbial species). After 7 days of storage, only the *Pseudomonas* counts showed the highest value in Ctr sample. The differences became more evident after 14 days of storage, then increased over time and regarded all the bacteria groups. Considering the *Enterobacteriaceae* 2.7 log CFU g^−1^ count were found in Ctr at the end of storage, vs. 0.9–1.2 log CFU g^−1^ in the other samples; a similar trend was observed for *Pseudomonas*, ranging from 1 to 1.4 log CFU g^−1^ in the experimental samples vs. 2 log CFU g^−1^ found in the Ctr. The comparison among experimental samples evidenced that the best outcomes were obtained in the case of a combination of the protective starter with MAP; the use of *Lactobacillus plantarum*, as a producer of bacteriocins, may have played a major role, as widely demonstrated in the literature [58,59,60]; however, a synergic effect with MAP seems to be evident. 

**Figure 4:**
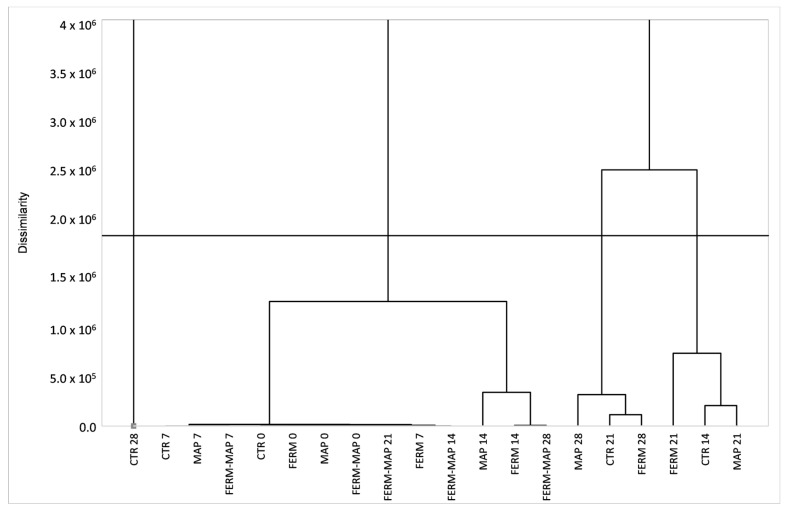
Agglomerative Hierarchical Clustering (AHC) based on dissimilarities of burrata samples volatile organic compounds profile.

The volatile organic compounds data was used to perform Agglomerative Hierarchical Clustering (AHC) based on dissimilarities. It is a clustering (or classification) method that allows to group of samples into clusters by the dendrogram, aiming to better observe the differences in the VOC profile evolution (Figure 4). The dotted line represents the truncation that generates four different clusters, in which burrata samples were separated by the evolution of the VOC profile during storage. The differences among samples’ VOC profiles were evident, showing Ctr as having the fastest VOC production process that, overall, penalizes the product.

The red cluster is more homogeneous than the others being dendrogram flatter; it represents the VOC profile of samples ranging from 0 to 14 days of storage (with some exceptions). This cluster grouped the products until 14 days of storage, but among them are also present the samples having 21 and 28 days of storage, which are Ferm-MAP. The second cluster, the purple one, grouped Ferm and MAP after 21 days of storage and Ctr after 14 days. This meant that the VOC profile of the latter was ascribable to the older experimental samples. Then, the same results were found within the green cluster showing Ctr after 21 days which clustered with MAP and Ferm after 28 days of storage. Finally, the fourth cluster (black one) is represented only by Ctr T28, the sample with the highest amount of VOC. Therefore, it is clear that among samples, Ctr was the richest in volatile compounds and the one that perishes more rapidly, being grouped with older experimental samples. The high VOC production is usually related to microbial growth [61].

On the contrary, the most amazing results were ascribable to the Ferm-MAP sample, which showed reduced VOC production compared to Ctr. Moreover, such a sample at days 21 and 28 clustered with samples within the Ctr producers’ shelf-life, theoretically highlighting a more delicate aroma than the Ctr sample. This result suggests a strong inhibition of volatile production by combining protective culture starter and MAP, leading to a less pronounced off-flavor. To sum up, the VOC profile of the Ctr sample after 14 and 21 days of storage was grouped with older experimental samples, while Ctr after 28 days was the worst sample, the richest in VOC compounds. The total VOC amount of Ctr at the end of the storage had a 3-fold higher concentration than the other samples (6641 µg kg^−1^ vs. 1467, 1925 and 653 µg kg^−1^ of Ferm, MAP and Ferm-MAP at T28, respectively). These results were better than those reported by Natrella et al. [25], in which samples after 21 days had a higher total VOC than that found in this work after 28 days of storage, which could reflect a higher off-flavor perception. After 28 days, the highest chemical classes found were alcohols and acids for all samples except for Ferm-MAP, in which the ketones class was the most representative.

**Figure 5:**
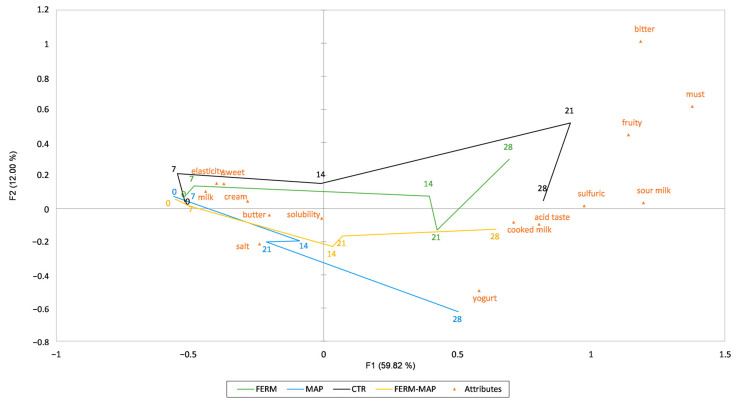
Multivariate analysis, Temporal Check All That Apply (TCATA) based on sensory results. Ctr—Control burrata; MAP—burrata packaged in the modified atmosphere; Ferm—burrata added of microbial protection culture; Ferm-MAP—burrata added of microbial protection culture and packaged in modified atmosphere.

The sensory results were subjected to multivariate analysis to summarize the evolution during the storage of the samples (Figure 5). The two components (PCs) of the plot explained 71.82% of the total variance. In general, the first PC explained most of the variance with 59.82%, in which it is possible to observe the separation among samples based on the storage time as a discrimination variable. In fact, sample worsening is visible from the left (fresh products) to the right (stored products). On the other hand, dissimilarity among the type of sample is shown along the second principal component. Better results were obtained by combining protective culture starter with MAP, which followed the negative side of the second PC in the bottom right quadrant, where better sensory descriptors were placed compared to the ones on the upper-right side. During the first week of storage, no differences were found among the samples—all remained fresh and similar to each other. Then, starting from days 14 to 28, the samples separate into space, with Ctr and Ferm upward on the positive side of PC2 and MAP and Ferm-MAP downward through the negative side. The differences among samples were based on the main sensory characteristic perceived by panelists, mostly after the two weeks of storage. Firstly Ctr, then Ferm at day 28 were described as having sulfuric and sour milk odor notes, while MAP and Ferm-MAP samples were characterized by notes of yogurt and cooked milk. Thus, milder descriptors were used to refer to the latter samples, even if, after 28 days, all samples were considered inedible. On the contrary, after 21 days, the MAP and Ferm-MAP samples were considered by the panelists to be very similar to the same sample after 14 days of storage. 

In sum, the overall characteristics decrease over time for almost all products (from the left to the right side of the plot); the panelists highlighted the worst quality of samples after 28 days of storage, while after 21 days, some samples still were acceptable, i.e., MAP packaged samples were similar to the same samples at T14, which still had good characteristics, and were very different from Ctr. These outcomes revealed that MAP alone or in combination with protective starter, whose bacteriocins have been demonstrated to have antimicrobial activity against a wide range of bacteria [58,59,60], better preserved the sensory characteristics of burrata in agreement with VOC and the other chemicals results.

## 4. Conclusions

The use of MAP in conjunction with bioprotective starter allowed for better preserve the chemical and sensory characteristics of burrata, which gained a few days of shelf life compared to the control (from 14 to 21 days). The combination of a modified atmosphere and protective starter allowed, during storage, a significant delay in lactose and citric acid degradation, which are directly linked to microbial growth. Accordingly, the microbial counts of undesired bacteria (*Enterobacteriaceae*, Coliforms, *Staphylococcus*, *Pseudomonas* spp.) were lower than control burrata as well as microbial metabolites such as organic acids, free fatty acids and volatile organic compounds, among which are more probable to find molecules responsible of off-flavors in this cheese. These results were confirmed by the sensory evaluation. In conclusion, the synergy between the modified atmosphere and bioprotective starter, in conjunction with the good quality of raw matter and good manufacturing practices, can significantly improve the microbiological stability of burrata without using chemical additives. Further investigation is needed to assess the effect of the application of modified atmosphere packaging in the presence of the governing liquid when it is technically possible.

## Figures and Tables

**Table 1:** Cell counts (Log CFU g^−1^ ± SD), the average value of three replicates. Different letters indicate the differences among samples (storage times were considered separately). *p* < 0.05.

LOG CFU g^−1^	Storage (Days)	*Enterobacteriaceae*	Coliforms	*E. Coli*	*Staphylococcus* Coagulase +	*Pseudomonas*
Ctr	T0	2.2 ± 0.1 ^A^	2.1 ± 0.1 ^A^	<1 ^A^	<1 ^A^	2.3 ± 0.1 ^A^
Ferm	T0	1.9 ± 0.1 ^B^	1.9 ± 0.1 ^B^	<1 ^A^	<1 ^A^	2.0 ± 0.1 ^BC^
MAP	T0	2.0 ± 0.1 ^AB^	2.0 ± 0.1 ^AB^	<1 ^A^	<1 ^A^	2.1 ± 0.1 ^AB^
Ferm MAP	T0	1.7 ± 0.2 ^C^	1.5 ± 0.1 ^C^	<1 ^A^	<1 ^A^	1.9 ± 0.1 ^C^
Ctr	T7	1.0 ± 0.1 ^B^	1.3 ± 0.2 ^C^	<1 ^A^	<1 ^A^	3.3 ± 0.1 ^A^
Ferm	T7	2.7 ± 0.3 ^A^	2.6 ± 0.2 ^AB^	<1 ^A^	<1 ^A^	3.0 ± 0.1 ^B^
MAP	T7	2.8 ± 0.2 ^A^	2.7 ± 0.1 ^A^	<1 ^A^	<1 ^A^	2.7 ± 0.1 ^C^
Ferm MAP	T7	2.6 ± 0.2 ^A^	2.5 ± 0.1 ^B^	<1 ^A^	<1 ^A^	2.5 ± 0.1 ^D^
Ctr	T14	1.0 ± 0.1 ^A^	1.0 ± 0.1 ^B^	<2 ^A^	<2 ^A^	3.5 ± 0.4 ^A^
Ferm	T14	0.7 ± 0.1 ^B^	2.9 ± 0.3 ^A^	<2 ^A^	<2 ^A^	3.0 ± 0.2 ^A^
MAP	T14	0.7 ± 0.1 ^B^	2.9 ± 0.3 ^A^	<2 ^A^	<2 ^A^	3.0 ± 0.3 ^A^
Ferm MAP	T14	0.3 ± 0.1 ^C^	2.9 ± 0.4 ^A^	<2 ^A^	<2 ^A^	2.9 ± 0.2 ^A^
Ctr	T21	1.7 ± 0.3 ^A^	1.7 ± 0.2 ^A^	<2 ^A^	<2 ^A^	3.6 ± 0.2 ^A^
Ferm	T21	0.9 ± 0.1 ^B^	0.8 ± 0.1 ^B^	<2 ^A^	<2 ^A^	3.3 ± 0.1 ^B^
MAP	T21	0.8 ± 0.2 ^BC^	0.7 ± 0.1 ^BC^	<2 ^A^	<2 ^A^	3.0 ± 0.2 ^B^
Ferm MAP	T21	0.6 ± 0.1 ^C^	0.6 ± 0.1 ^C^	<2 ^A^	<2 ^A^	2.9 ± 0.3 ^B^
Ctr	T28	2.7 ± 0.3 ^A^	2.6 ± 0.2 ^A^	<3 ^A^	<2 ^A^	2.0 ± 0.2 ^A^
Ferm	T28	1.2 ± 0.1 ^B^	1.1 ± 0.1 ^B^	<2 ^B^	<2 ^A^	1.4 ± 0.2 ^B^
MAP	T28	1.1 ± 0.1 ^BC^	1.0 ± 0.1 ^BC^	<2 ^B^	<2 ^A^	1.2 ± 0.1 ^B^
Ferm MAP	T28	0.9 ± 0.1 ^C^	0.9 ± 0.1 ^C^	<2 ^B^	<2 ^A^	1.0 ± 0.1 ^C^

## Data Availability

Data are contained within the article.
